# Does Global Warming Increase Establishment Rates of Invasive Alien Species? A Centurial Time Series Analysis

**DOI:** 10.1371/journal.pone.0024733

**Published:** 2011-09-08

**Authors:** Dingcheng Huang, Robert A. Haack, Runzhi Zhang

**Affiliations:** 1 CAS Key Laboratory of Zoological Systematics and Evolution, Institute of Zoology, Chinese Academy of Sciences, Beijing, China; 2 Graduate School of Chinese Academy of Sciences, Beijing, China; 3 USDA Forest Service, Northern Research Station, East Lansing, Michigan, United States of America; 4 State Key Laboratory of Integrated Management of Pest Insects and Rodents, Institute of Zoology, Chinese Academy of Sciences, Beijing, China; Institute of Marine Research, Norway

## Abstract

**Background:**

The establishment rate of invasive alien insect species has been increasing worldwide during the past century. This trend has been widely attributed to increased rates of international trade and associated species introductions, but rarely linked to environmental change. To better understand and manage the bioinvasion process, it is crucial to understand the relationship between global warming and establishment rate of invasive alien species, especially for poikilothermic invaders such as insects.

**Methodology/Principal Findings:**

We present data that demonstrate a significant positive relationship between the change in average annual surface air temperature and the establishment rate of invasive alien insects in mainland China during 1900–2005. This relationship was modeled by regression analysis, and indicated that a 1°C increase in average annual surface temperature in mainland China was associated with an increase in the establishment rate of invasive alien insects of about 0.5 species year^−1^. The relationship between rising surface air temperature and increasing establishment rate remained significant even after accounting for increases in international trade during the period 1950–2005. Moreover, similar relationships were detected using additional data from the United Kingdom and the contiguous United States.

**Conclusions/Significance:**

These findings suggest that the perceived increase in establishments of invasive alien insects can be explained only in part by an increase in introduction rate or propagule pressure. Besides increasing propagule pressure, global warming is another driver that could favor worldwide bioinvasions. Our study highlights the need to consider global warming when designing strategies and policies to deal with bioinvasions.

## Introduction

Establishment rates of invasive alien species ( =  number of new invasive alien species discovered or reported per annum for a recipient region [1]) have been increasing in China [2], [3], Europe [1], [4], [5] and North America [6], [7] in recent decades. These trends are widely attributed to increased rates of species introductions associated with increasing international trade [1], [7]–[9], but rarely linked to environmental changes such as global warming that can directly or indirectly influence establishment success of newly-introduced alien species in their recipient regions [10]–[13]. Considerable efforts have been made to prevent new introductions as well as manage already established alien species [14]–[16], but new invasions are continually being reported [1], [3]. To better understand bioinvasions and develop more effective strategies to slow or prevent them, it is crucial to understand the relationship between bioinvasions and environmental change.

As a significant component of climate change, rising ambient temperature is an important consideration, especially for temperature-sensitive invaders such as insects. Recent reviews suggest that global warming could facilitate bioinvasions across all steps of the invasion process including species introduction, colonization, establishment and spread [11], [13]. The recent rapid expansion of the palm *Trachycarpus fortunei* (Hook.) H. Wendl. in Europe is a prominent example, demonstrating that global warming can provide opportunities for alien species to become established in areas that were not once suitable [17]. Similarly, warmer temperatures increase the flight activity of *Thaumetopoea pityocampa* (Denis and Schiffermüller) female moths, enabling them to disperse over greater distances [18].

Our study reports finding a positive relationship between the establishment rates of invasive alien insects (IAIs) and changes in average annual surface air temperature in mainland China (referred to hereafter as China), the United Kingdom, and the contiguous United States (referred to hereafter as the United States). The aim of our study is to better understand the role of global warming in the increasing rate of IAI establishments worldwide during recent decades. We focused on insects because they are a major group of invasive species, they are well known taxonomically, and they represent a typical group of poikilothermic animals and thus should be sensitive to changes in ambient temperature.

## Materials and Methods

### Data collection

Establishment rate (unit: species year^−1^) was calculated using the first-year an IAI was recorded or reported within the recipient region during 1900–2005 (inclusive). To build this series for China, we compiled a list of IAIs and their first-recorded dates of occurrence in China from professional literature, particularly from checklists of invasive alien species and journal articles.

Additional data were obtained for the United Kingdom and the continental United States mostly from professional publications. Information described as “date of introduction”, “arrival date”, or “year of first record” was considered to represent the year of establishment [19]–[22]. However, we recognize that the year of establishment often occurs many years before the year of first record. When the time of first record was described as a decade then we entered the midpoint of the decade (e.g., 1930s was entered as 1935), and when the time was reported as prior to a specific year then we entered that year (e.g., prior to 1940 was entered as 1940) [20].

Overall, we collected data for 54 IAIs that are of economical and environmental importance in China (Table S1), for 296 invasive and non-invasive alien insects in the United Kingdom (Table S2), and for all 44 invasive and non-invasive alien bark and ambrosia beetles (Coleoptera: Scolytinae) recognized as established in the continental United States during 1900–2005 (Table S3). To minimize potential confounding effects from human activities, we excluded species introduced intentionally into the three study regions. Next, we counted the number of new IAIs recorded each year and calculated the establishment rate for each region. An 11-year moving average of IAIs (time span: 1905–2000) was then calculated from the series of annual values for each study region (Table S4 provides summary data for the response and explanatory variables used in this study).

Our explanatory variable, annual change in average annual surface air temperature (unit: °C year^−1^) in China, the United Kingdom and the United States were calculated using data derived from references [23], [24] and [25], respectively. For the United Kingdom and United States, updated datasets are publicly available at http://www.metoffice.gov.uk and http://data.giss.nasa.gov, respectively. The years covered by these datasets are 1873–2005 for China, 1659–2011 for the United Kingdom, and 1880–2011 for the United States. We adjusted all raw data relative to 1961–1990 for each country, which is a widely-used reference period [26], [27]. Next, we took an 11-year moving average of temperature for each country during the period 1905–2000, and used the resulting series as a metric to explore for trends along with the 11-year moving average of IAIs over the same time period.

We also explored the relationships between changes in average annual surface air temperature and establishment rate of IAIs after accounting for changes in levels of international trade during the period 1950–2005. For trade data, we used the International Financial Statistics of imported merchandise (unit: million US$ year^−1^) for the three study regions during the years 1950–2005. The years covered by these datasets are 1950–2010 for China, and 1948–2010 for the United Kingdom and United States. These data are publically available at http://www.imf.org/external/data.htm.

### Statistics

Cross-correlation function (CCF) was used to identify potential associations between the establishment rate of IAIs and changes in temperature in each country. Linear least-square regression analysis was conducted to model the relationship for each country. We used the following equation that links establishment rate of IAIs (*r*) in year *t* to temperature change (*h*) in year *t*.
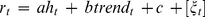
(1)

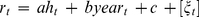
(2)where *c* represents fixed effects accounting for time-invariant characteristics that might explain differences in the baseline level of bioinvasion risk, *btrend_t_* suggests a common time trend of bioinvasion risk associated with the growth of human activities, *byear_t_* reflects a region-specific time trend used to control for variables that could evolve over time (e.g., international trade) and thereby alter the risk of bioinvasion, and [ξ*_t_*] is the residual error term. We first ran model [1] using only *btrend_t_* to determine the effect of global warming without the variable *byear_t_*, and then we ran model [2] including international trade without the variable *btrend_t_*.

## Results

**Figure 1:**
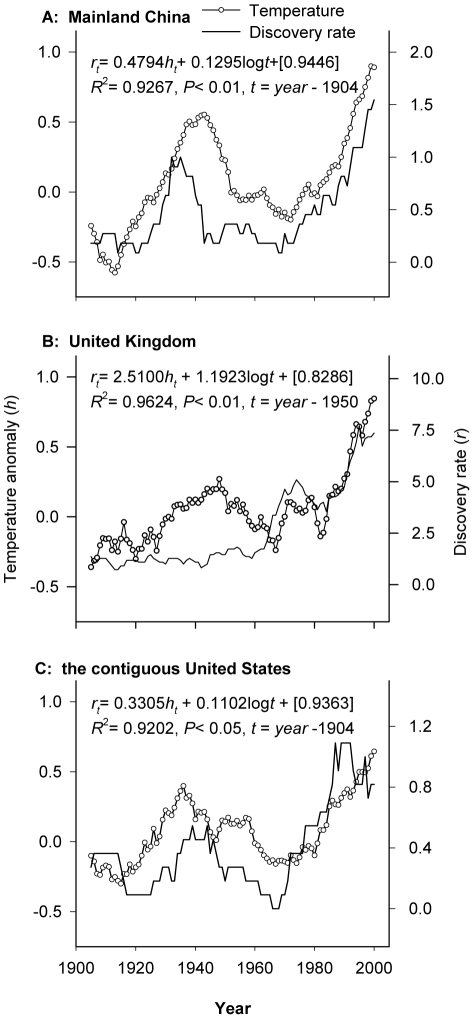
Associations between establishment rates of invasive alien insects and average annual surface air temperature changes during 1900–2005. A: Mainland China; B: the United Kingdom; C: the contiguous United States. Temperature change (calculated as the deviation from the 1961–1990 mean) and establishment rate are presented as an 11-year moving average. The establishment rate (*r*) as a function of temperature change (*h*) was modeled using linear least squares regression with a common time series. The numbers in the square brackets are the regression residuals. See text for details.

CCF analysis detected a significant positive relationship between the establishment rate of IAIs and annual mean temperature change in China during the study period 1905–2005 (Table 1). All CCF values were significant (*P*<0.05) when testing the model with time lags of 0 to 5 years. All analyses using the 11-year moving average data for temperature and IAIs produced higher CCF values compared with the analyses where annual values were used (Table 1). The close association between the 11-year moving average for temperature and IAIs in China can be observed in Fig. 1A, and was well described by a linear equation with common time trend (Fig. 1A). This equation predicts that a 1°C increase in mean annual temperature corresponds to an increase of about 0.5 IAI species year^−1^ in China. The relationship between increasing average annual surface air temperature and establishment rate of IAIs remained significant after inclusion of the annual value of imported merchandise in the regression models during 1951–2005, using either the 11-year moving average time series or simply the annual values (Table 2). Similar significant associations were noted between establishment rates of alien insect species and changes in average annual surface air temperature in the United Kingdom and the United States (Fig. 1B-C, Tables 1,2), suggesting again that increasing establishment rates of IAIs may be related to increasing ambient temperature.

**Table 1:** Cross-correlation function values for the relationship between establishment rates of invasive alien insects and temperature change in mainland China (CN), the United Kingdom (UK), and the contiguous United States (US).

Pair	Time lag (year)	CCF1	CCF2
*r*_CN_ ∼ *h*_CN_	0	0.4317*	0.7657*
	1	0.2590*	0.6965*
	2	0.3170*	0.6234*
	3	0.3071*	0.5512*
	4	0.2673*	0.4817*
	5	0.2435*	0.4164*
*r*_UK_ ∼ *h*_UK_	0	0.3745*	0.7201*
	1	0.3973*	0.6554*
	2	0.3464*	0.5830*
	3	0.2303*	0.5147*
	4	0.1732*	0.4539*
	5	0.1759*	0.4021*
*r*_US_ ∼ *h*_US_	0	0.3261*	0.6666*
	1	0.3180*	0.6338*
	2	0.1717	0.5895*
	3	0.1999*	0.5476*
	4	0.2815*	0.5002*
	5	0.1812	0.4481*

Establishment rate and temperature change (deviation from the 1961–1990 mean) were indicated by *r* and *h*, respectively. In CCF1, *r* and *h* are based on annual data during the period 1905–2005, while in CCF2, *r* and *h* are based on 11-year moving-averages.

Asterisks (*) indicate that the coefficients were significant (2-tailed): *P*<0.05.

**Table 2:** Effect of changes in average annual surface air temperature and level of international trade on the establishment rate of invasive alien insects in mainland China, the United Kingdom, and the contiguous United States during 1951–2005.

	Temperature		Imports		*R* ^2^
	CE	SE	CE	SE	
**China**					
Model 1	0.8792***	0.0741	0.0728***	0.0138	0.9651
Model 2	1.0679***	0.0749	0.0424***	0.0026	0.6183
**United Kingdom**					
Model 1	1.6961*	0.7715	0.4491***	0.1144	0.9660
Model 2	1.8133*	0.6304	0.3620***	0.0343	0.2786
**United States**					
Model 1	0.5527*	0.2826	0.0422***	0.0100	0.8590
Model 2	0.5527*	0.2604	0.0435***	0.0105	0.1408

In these regression analyses, establishment rate of invasive alien insects was used as the response variable, temperature change (deviation from the 1961–1990 mean) and international trade (annual value of imported merchandise) were the explanatory variables. For model 1, the variables were expressed as an 11-year moving-average time series; and for model 2, the variables were expressed as an annual time series. In the regression for each country, import data were log transformed. CE and SE mean regression co-efficient and its standard error, respectively. Asterisks indicate that the coefficients were significant (2-tailed):

*P<0.05;

**P<0.01,

***P<0.001.

## Discussion

The discovery of newly established alien species depends on many factors such as the temporal pattern of alien introductions, relative abundance or size of the founding populations, and the sampling efforts by humans [8]. Increasing foreign trade, along with a concomitant increase in the propagule pressure of alien species, is another important determinant of species introductions [1], [7], [9], [28].

We found significant positive associations between establishment rates of IAIs and changes in mean annual surface air temperature in our three study regions over the past century. Such results suggest that rising ambient temperatures have the potential to increase establishment rates of IAIs. Warmer temperatures can favor establishment of alien insects both directly and indirectly [10], [29]–[33]. For example, warmer temperatures can provide new areas for establishment of IAIs that were previously unsuitable [34], enable insects to shift their geographic range polewards [35], and to cross barriers that previously limited their natural ranges [10]. In addition, warmer temperatures can hasten insect growth and reproduction [29], improve winter survival [30], allow for greater multivoltinism [36] and higher population densities [37], [38], and increase plant susceptibility and suitability to herbivorous insects [39]. Consequently, these favorable factors could lead to higher rates of establishments, shorter latent periods (i.e. time lag between introduction and discovery), and higher probabilities of population outbreaks.

Moreover, our results showed that the effects of increasing average annual surface air temperature on establishment rate were sufficiently robust to remain significant even when adjusted for changes in international trade, indicating that establishment rate of IAIs can increase even when there is no increase in propagule pressure. This can occur, in part because (1) not all alien species are introduced by human activity, but rather some species arrive in new areas because of natural range expansion [35], [40], [41], and (2) warmer temperatures can allow more introduced species to become established.

It is important to recognize that IAI establishment rate can be influenced by factors other than propagule pressure and global warming. These other factors could include biotic traits and variation in the degree of invasiveness of the introduced species [42], [43], influence of human disturbance on the invasibility of the recipient regions [43]–[45], and precipitation chemistry and other aspects of climate change [46], [47].

In addition, increasing the effort to survey for new IAIs would be expected to result in more discoveries of new alien species. Examining this hypothesis would be valuable but is beyond the scope of the present paper. However, it should be noted that the sampling efforts in China during the 1930s–1940s, a period of general warfare, could be expected to have been lower than during the more peaceful years of the 1950s–1960s. However, it is interesting to note that many more IAIs were discovered in China during the in 1930s–1940s than during the 1950s–1960s (Fig. 1A). This example confirms the theoretical prediction that the expected establishment curve can increase even when both propagule pressure and survey effort remain nearly constant [8].

In conclusion, our study suggests that the increase in establishment rates of IAIs in China, the United Kingdom, and the United States during the past century can be partially explained by global warming given that warmer temperatures can facilitate bioinvasions worldwide [13], [47]. Moreover, our findings suggest that the interaction between global warming and bioinvasion should be considered by plant health protection specialists and policy makers.

## Supporting Information

Table S1 **List of invasive alien insects and their first-recorded dates of establishment in mainland China during 1900–2005 (inclusive).**
(DOC)

Table S2 **List of invasive and noninvasive alien insects and their first-recorded dates of establishment in the United Kingdom during 1900–2005 (inclusive).**
(DOC)

Table S3 **List of invasive and noninvasive alien scolytines and their first-recorded dates of establishment in the contiguous United States during 1900–2005 (inclusive).**
(DOC)

Table S4 **Minimum and maximum values for the response and explanatory variables used in the present study by country.**
(DOC)
