# Supplementary material for: Does Global Warming Increase Establishment Rates of Invasive Alien Species? A Centurial Time Series Analysis
Source: PLoS One. 2011 Sep 8;6(9):e24733. doi: 10.1371/journal.pone.0024733 (PMC3169637; doi:10.1371/journal.pone.0024733)
Supplement: Table S1 — List of invasive alien insects and their first-recorded dates of establishment in mainland China during 1900–2005 (inclusive). (DOC) [file pone.0024733.s001.doc]

# Table S1. List of invasive alien insects and their first-recorded dates (FRD) in mainland China during 1900–2005 (inclusive).

| **ID** | **Scientific name** | **FRD** | **Source** |
| --- | --- | --- | --- |
|  | **Blattodea** |  |  |
|  | **Blattellidae** |  |  |
| 1 | *Blattella germanica* (Linnaeus, 1767) | 1935 | 1 |
|  | **Blattidae** |  |  |
| 2 | *Periplaneta americana* (Linnaeus, 1758) | 1931 | 2 |
| 3 | *Periplaneta australasiae* (Fabricius, 1775) | 1931 | 3 |
|  | **Coleoptera** |  |  |
|  | **Bostrychidae** |  |  |
| 4 | *Heterobostrychus aequalis* (Waterhouse, 1884) | 1988 | 4 |
|  | **Brentidae** |  |  |
| 5 | *Cylas formicarius* Olivier, 1807 | 1965 | 5 |
|  | **Bruchidae** |  |  |
| 6 | *Acanthoscelides macrophthalmus* (Schaeffer, 1907) | 1999 | 6 |
| 7 | *Acanthoscelides obtectus* (Say, 1859) |  |  |
| 8 | *Bruchus pisorum* (Linnaeus, 1758) | 1950s | 4 |
| 9 | *Bruchus rufimanus* Boheman, 1833 | 1937 | 7 |
| 10 | *Callosobruchus maculatus* (Fabricius, 1775) | 1997 | 8 |
| 11 | *Zabrotes subfasciatus* (Boheman, 1833) | 1975 | 9 |
|  | **Buprestidae** |  |  |
| 12 | *Agrilus mali* Matsumura, 1924 | 1934 | 10 |
|  | **Chrysomelidae** |  |  |
| 13 | *Brontispa longissima* (Gestro, 1885) | 1999 | 11 |
| 14 | *Leptinotarsa decemlineata* (Say, 1824) | 1993 | 4 |
| 15 | *Ophraella communa* LeSage, 1986 | 2004 | 12 |
|  | **Curculionidae** |  |  |
| 16 | *Axionicus insignis* Pascoe, 1869 | 2002 | 13 |
| 17 | *Dendroctonus valens* LeConte, 1859 | 1998 | 4 |
| 18 | *Lissorhoptrus oryzophilus* Kuschel, 1952 | 1988 | 4 |
| 19 | *Rhabdoscelus lineaticollis* (Heller) | 2002 | 14 |
| 20 | *Rhynchophorus ferrugineus* Herbst, 1795 | 1997 | 15 |
| 21 | *Sitophilus granarius* (Linnaeus, 1758) | 1954 | 16 |
|  | **Dermestidae** |  |  |
| 22 | *Trogoderma granarium* Everts, 1898 | 1962 | 4 |
|  | **Hispidae** |  |  |
| 23 | *Octodonta nipae* (Maulik, 1921) | 2001 | 17 |
|  | **Diptera** |  |  |
|  | **Agromyzidae** |  |  |
| 24 | *Liriomyza bryoniae* (Kaltenbach, 1858) | 1985 | 18 |
| 25 | *Liriomyza huidobrensis* Blanchard, 1926 | 1993 | 15 |
| 26 | *Liriomyza sativae* Blanchard, 1938 | 1993 | 2 |
| 27 | *Liriomyza trifolii* (Burgess, 1880) | 2005 | 19 |
|  | **Cecidomyiidae** |  |  |
| 28 | *Contarinia sorghicola* Coquillett, 1899 |  |  |
| 29 | *Mayetiola destructor* Say, 1817 | 1980 | 20 |
| 30 | *Obolodiplosis robiniae* Haldeman, 1847 | 2004 | 21 |
|  | **Tephritidae** |  |  |
| 31 | *Bactrocera cucurbitae* Coquillett, 1899 |  |  |
| 32 | *Bactrocera dorsalis* Hendel, 1912 | 1934 | 22 |
| 33 | *Carpomya vesuviana* Costa, 1854 | 2008 | 23 |
|  | **Hemiptera** |  |  |
|  | **Aleyrodidae** |  |  |
| 34 | *Aleurodicus dispersus* Russell, 1965 | 2006 | 24 |
| 35 | *Bemisia argentifolii* Bellows and Perring, 1994 | 1994 | 25 |
| 36 | *Bemisia tabaci* (Gennadius, 1889) | 1949 | 4 |
| 38 | *Trialeurodes vaporariorum* (Westwood, 1856) | 1975 | 1 |
| 37 | *Bemisia tabaci* (Gennadius, 1889) Q biotype | 2003 | 26 |
|  | **Aphididae** |  |  |
| 39 | *Eriosoma lanigerum* (Hausmann, 1802) | 1914 | 27 |
|  | **Diaspididae** |  |  |
| 40 | *Hemiberlesia pitysophila* Takagi, 1969 | 1982 | 4 |
| 41 | *Diaspidiotus perniciosus* (Comstock, 1881) | 1930 | 10 |
|  | **Margarodidae** |  |  |
| 42 | *Matsucoccus matsumurae* (Kuwana, 1905) | 1942 | 28 |
| 43 | *Icerya aegyptiaca* (Douglas, 1890) | 1908 | 29 |
| 44 | *Icerya purchasi* Maskell, 1879 | 1908 | 10 |
|  | **Phylloxeridae** |  |  |
| 45 | *Daktulosphaira vitifoliae* (Fitch, 1855) | 1892 | 4 |
|  | **Pseudococcidae** |  |  |
| 46 | *Oracella acuta* (Lobdell, 1930) | 1990 | 4 |
| 47 | *Phenacoccus solenopsis* Tinsley, 1898 | 2008 | 30 |
| 48 | *Dysmicoccus neobrevipes* Beardsley, 1959 | 1998 | 31 |
|  | **Scutelleridae** |  |  |
| 49 | *Eurygaster integriceps* Puton, 1881 |  | 32 |
|  | **Tingidae** |  |  |
| 50 | *Corythucha ciliata* (Say, 1832) | 2006 | 33 |
|  | **Hymenoptera** |  |  |
|  | **Eulophidae** |  |  |
| 51 | *Leptocybe invasa* Fisher and LaSalle, 2004 | 2007 | 34 |
| 52 | *Quadrastichus erythrinae* Kim, 2004 | 2005 | 35 |
|  | **Formicidae** |  |  |
| 53 | *Anoplolepis gracilipes* (Smith, 1857) |  |  |
| 54 | *Pheidole megacephala* (Fabricius, 1793) |  |  |
| 55 | *Solenopsis invicta* Buren, 1972 | 2004 | 36 |
|  | **Tenthredinidae** |  |  |
| 56 | *Nematus melanaspis* Hartig, 1840 | 1986 | 37 |
|  | **Isoptera** |  |  |
| 57 | *Incisitermes minor* (Hagen, 1858) | 1937 | 2 |
|  | **Lepidoptera** |  |  |
|  | **Arctiidae** |  |  |
| 58 | *Hyphantria cunea* (Drury, 1773) | 1979 | 38 |
|  | **Carposinidae** |  |  |
| 59 | *Carposina niponensis* Walsingham, 1900 | 1927 | 39 |
|  | **Gelechiidae** |  |  |
| 60 | *Pectinophora gossypiella* (Saunders, 1844) | 1940 | 10 |
| 61 | *Phthorimaea operculella* (Zeller, 1873) | 1937 | 40 |
|  | **Lymantriidae** |  |  |
| 62 | *Lymantria dispar dispar* (Linnaeus, 1758) | 1920 | 10 |
|  | **Tineidae** |  |  |
| 63 | *Opogona sacchari* Bojer, 1856 | 1997 | 4 |
|  | **Tortricidae** |  |  |
| 64 | *Cydia pomonella* Linnaeus, 1758 | 1953 | 41 |
| 65 | *Grapholitha inopinata* Heinrich 1928 | 1937 | 10 |
|  | **Thysanoptera** |  |  |
|  | **Thripidae** |  |  |
| 66 | *Frankliniella occidentalis* (Pergande, 1895) | 2003 | 42 |

List of FRD information sources:

1. Xu Z, Chen W, Cai G (2008) Identification and control of invasive alien species in Hangzhou disctrict. Hangzhou: Zhejiang University Press. pp189.

2. Li Z, Xie Y (2002) Invasive Alien Species in China. Beijing: China Forestry Publishing House. pp 211.

3. Zhang S, Zhao Y (1996) Geographical Distribution of Agricultural and Forestry Insects in China. Beijing: China Agricultural Press. pp 400.

4. Xu H, Qiang S (2004) Checklist of invasive alien species in China. Beijing: China Environmental Science Press. pp 432.

5. Zhong P (1994) The occurrence of *Cylas formicarius* in Zhejiang and its quarantine approaches. Plant Quarantine 8: 304–305.

6. Qin X, Zhang S, Zhang Z, Chen Z, Tang J, et al. (2007) A new invasive insect pest *Acanthoscelides macrophthalmus* (Coleoptera: Bruchidae). Chin J Tropic Crops 28: 101–103.

7. Kuoh JL (1952) A preliminary study of the horse bean weevil. Acta Entomol Sin 2: 38–46.

8. Ding J, Mack RN, Lu P, Ren M, Huang H (2008) China's booming economy is sparking and accelerating biological invasions. BioScience 58: 317–324.

9. Zhu W, Deng Y (1991) A preliminary study on the biology of *Zabrotes subfasciatus* (Bruchidae, Coleoptera). J Southwest Agric Univ 13: 243–246.

10. Hong X, Xu H, Li H, Xie L (2003) Alien invasive insects and pathogens in Jiangsu Province: current status, influence and control. J Nanjing Agric Univ 26: 116–123.

11. Zhang Z, Cheng D, Jiang D, Xu H (2004) Spread, damage and control methods of *Brontispa longissima*. Entomol Knowl 41: 522–526.

12. Meng L, Li B (2005) Advances on biology and host specificity of the newly introduced beetle, *Ophraella communa* Lesage (Coleoptera: Chrysomelidae), attacking *Ambrosia artemisiifollia* (Compositae) in continent of China. Chin J Biol Control 21: 65–69.

13. Li H, Han H, Zhang R, Xue D (2005) List of invasive alien insects in mainland China. In: Qiao G, Chen H, Xiao H, editors. Research Advance in Entomology. Beijing. pp. 10–17.

14. Wang G, Chen J, Han R (2005) Advances in the research on the biology and control of coconut weevil *Rhabdwscelus lineaticollis* (Heller). Nat Enemies Insects 27: 127–133.

15. Wan F, Zheng X, Guo J (2005) Biology and management of invasive alien species in agriculture and forestry. Beijing: Science Press. pp 820.

16. Li L, Lu L, Liao S (1961) Discovery of *Sitophilus grannaria* (L.) in Szechuan. Acta Entomol Sin 15: 19.

17. Sun J, Yu P, Zhang Y, Wang X (2003) A new invasive coconut pest in Hainan Province. Entomol Knowl 40: 286–287.

18. Wu J, Zhang W, Zeng L, Liang G (1997) Merits attention on a vegetable pest, *Liriomyza bryoniae* (Kaltenbach). Entomol Knowl 34: 216–218.

19. Wang X, Huang D, Li H, Xue D, Zhang R, et al. (2006) Invasion and identification of *Liriomyza trifolii* and its potential distribution areas in China. Chin Bull Entomol 43: 540–545.

20. Liang Y (1980) Discovery of *Mayetiola destructor* in northwestern China. Plant Quarantine 6: 2.

21. Yang Z, Qiao X, Bu W, Yao Y, Xiao Y, et al. (2006) First discovery of an important invasive insect pest, *Obolodiplosis robiniae* (Diptera: Cecidomyiidae) in China. Acta Entomol Sin 49: 1050–1053.

22. Zia Y (1937) Study on the Trypetidae of fruit-flies of China. Sinenia 8: 103–217.

23. Adili S, He S, Tian C, Luo Y, Yu F, et al. (2008) The ocurrence and pupae distribution pattern of *Carpomya vesuviana* in Turfan area. Plant quarantine 22: 295–297.

24. Yu G, Zhang G, Peng Z, Liu K, Fu Y (2007) The spiralling whitefly, *Aleurodicus dispersus*, invaded Hainan Island of China. Chin Bull Entomol 44: 428–431.

25. Liu S (2007) Invasion biology and sustainable management of *Bemisia tabaci*. In: Li D, Wu C, Wu Y, Meng X, editors. Proceedings of the 8th National Congress and Annual Meeting of the Entomological Society of China. Beijing: China Agricultural science and technology press. pp 629.

26. Chu D, Zhang Y, Brown JK, Cong B, Xu B, et al. (2006) The introduction of the exotic Q biotype of *Bemisia tabaci* from the mediterranean region into China on ornamental crops. Fla Entomol 89: 168–174.

27. Sun L, Tan X, Zhou H, Gu S, Guo J, et al. (2008) Investigation methods for population size of *Eriosoma lanigerum* in the orchard. Chin Bull Entomol 45: 818–822.

28. Zhao S, Chang G, Dang Z (1990) The occurrence and control strategy of *Matsucoccus matsumurae* in China. For Sci Technol 19: 1–3.

29. Zhao G, Zhou W, Wu Y (1994) Nonnative plant pathogens and insect pests in Taiwan. Taiwan Agric Res 11: 25–27.

30. Wu S, Zhang R (2009) A new invasive pest, *Phenacoccus solenopsis*, threatening seriously to cotton production. Chin Bull Entomol 46: 159–162.

31. Zhang X, Chen Z, Zhong Y, Wu H (2008) Elementary study on the life habits of *Dysmicoccus neobrevipes* (Beardsley). Entomol J East Chin 17: 22–25.

32. Zhang G, Fu W, Liu K (2008) Major Invasive Alien Species in Agricultural. Beijing: Science Press. pp 409.

33. Li C, Xia W, Wang F (2007) First records of *coryithucha ciliata* in China (Hemiptera, Tingidae). Acta Zootaxonomica Sinica 32: 944–946.

34. Wu Y, Jiang X, Li D, Luo J, Zhou G, et al. (2009) *Leptocybe invasa*, a new invasive forest pest making galls on twigs and leaves of *Eucalyptus* Trees in China (Hymenoptera: Eulophidae). Sci Silvae Sin 45: 161–163.

35. Yang W, Yu D, Jiao Y, Chen Z, Yang X (2005) First report of a new invasive pest, *Quadrastichus erythrinae* Kim in China. Plant Prot 31: 93.

36. Zeng L, Lu Y, He X, Zhang W, Liang G (2005) Identification of red imported fire ant *Solenopsis invicta* to invade mainland China and infestation in Wuchuan, Guangdon. Chin Bull Entomol 42: 144–148.

37. Qin Q, Miao Z, Tong Y (1994) A preliminary study on the biology of *Nematus melanaspis* Hartig. For Pest and Dis 13: 22–23.

38. Chen Z, Zhang S, Li Y, Wang J, Ai D (1980) Fall webworm (*Hyphantria cunea* Drury): An invasive pest newly introduced into China. Plant Prot 6: 25.

39. Liu Y, Cheng J, Mu J (1997) Review of the advances of the peach fruit-borer (*Carposina sasakii* Matsmura). J Shandong Agricl Univ 28: 207–214.

40. Li X, Jin X, Li Z (2005) The present status and developing tendency in *Phthorimaea operculella* research. J Qinghai Norm Univ (Nat Sci Ed) 27: 67–70.

41. Chang H (1953) Taxonomic notes on the codling moth, *Carpocapsa pomonella* L. in Sinkiang. Acta Entomol Sin 7: 467–472.

42. Zhang Y, Wu Q, Xu B, Zhu G (2003) The occurrence and damage of *Frankliniella occidentalis* (Thysanoptera: Thripidae) in Beijing. Plant Prot 23: 58–59.
