# Supplementary material for: Does Global Warming Increase Establishment Rates of Invasive Alien Species? A Centurial Time Series Analysis
Source: PLoS One. 2011 Sep 8;6(9):e24733. doi: 10.1371/journal.pone.0024733 (PMC3169637; doi:10.1371/journal.pone.0024733)
Supplement: Table S2 — List of invasive and noninvasive alien insects and their first-recorded dates of establishment in the United Kingdom during 1900–2005 (inclusive). (DOC) [file pone.0024733.s002.doc]

# Table S2. List of alien insects and their first-recorded dates (FRD) in the United Kingdom during 1900–2005 (inclusive).

| **ID** | **Scientific name** | **FRD** | **Source** |
| --- | --- | --- | --- |
|  | **Coleoptera** |  |  |
|  | **Anthicidae** |  |  |
| 1 | *Sticticomus tobias* (Marseul, 1879) | 2004 | 1 |
|  | **Carabidae** |  |  |
| 2 | *Amara cursitans* Zimmermann, 1832 | 1956 | 1 |
| 3 | *Leistus rufomarginatus* (Duftschmid, 1812) | 1942 | 1 |
| 4 | *Pterostichus quadrifoveolatus* Letzner, 1852 | 1900 | 1 |
| 5 | *Trechicus nigriceps* (Dejean, 1831) | 1903 | 1 |
| 6 | *Trechus subnotatus* Dejean, 1831 | 1950 | 1 |
|  | **Cerambycidae** |  |  |
| 7 | *Anoplophora chinensis* (Forster, 1771) | 2005 | 2 |
| 8 | *Trinophylum cribratum* Bates, 1878 | 1940 | 1 |
|  | **Chrysomelidae** |  |  |
| 9 | *Chrysolina americana* (Linnaeus, 1758) | 1963 | 1 |
| 10 | *Chrysolina viridana* (Küster, 1844) | 1961 | 1 |
| 11 | *Diabrotica virgifera virgifera* LeConte, 1858 | 2003 | 1, 3 |
| 12 | *Leptinotarsa decemlineata* (Say, 1824) | 1947 | 1 |
| 13 | *Luperomorpha xanthodera* (Fairmaire, 1888) | 2003 | 3 |
|  | **Cleridae** |  |  |
| 14 | *Paratillus carus* (Newman, 1840) | 1933 | 1 |
|  | **Coccinellidae** |  |  |
| 15 | *Harmonia axyridis* (Pallas, 1773) | 2004 | 1, 3, 4 |
| 16 | *Henosepilachna argus* (Geoffroy, 1762) | 1997 | 3 |
|  | **Cryptophagidae** |  |  |
| 17 | *Caenoscelis subdeplanata* Brisout de Barneville, 1882 | 1965 | 1 |
|  | **Curculionidae** |  |  |
| 18 | *Brachyderes incanus* (Linnaeus, 1758) | 1994 | 3 |
| 19 | *Crypturgus subcribrosus* Eggers, 1933 | 1986 | 1, 3 |
| 20 | *Dendroctonus micans* (Kugelann, 1794) | 1973 | 3 |
| 21 | *Diaprepes abbreviatus* (Linnaeus, 1758) | 1996 | 3 |
| 22 | *Euophryum confine* (Broun, 1881) | 1942 | 1 |
| 23 | *Euophryum rufum* (Broun, 1880) | 1934 | 1 |
| 24 | *Furcipus rectirostris* (Linnaeus, 1758) | 1979 | 1 |
| 25 | *Ips cembrae* (Heer, 1836) | 1955 | 1 |
| 26 | *Ips sexdentatus* (Börner, 1776) | 1919 | 1 |
| 27 | *Lixus scabricollis* Boheman, 1842 | 1992 | 3 |
| 28 | *Magdalis memnonia* (Gyllenhal, 1837) | 1971 | 1 |
| 29 | *Otiorhynchus armadillo* (Rossi, 1792) | 1998 | 1, 3 |
| 30 | *Otiorhynchus aurifer* Boheman, 1843 | 1978 | 1, 3 |
| 31 | *Otiorhynchus coecus* Germar, 1824 | 1950 | 1 |
| 32 | *Otiorhynchus crataegi* Germar, 1824 | 1985 | 1, 3 |
| 33 | *Otiorhynchus salicicola* Heyden, 1908 | 2000 | 1, 3 |
| 34 | *Otiorhynchus setosulus* Stierlin, 1861 | 1992 | 1, 3 |
| 35 | *Pachyrhinus mustela* (Herbst, 1797) | 1994 | 3 |
| 36 | *Scolytus laevis* Chapuis, 1869 | 1981 | 1, 3 |
| 37 | *Scolytus pygmaeus* (Fabricius, 1787) | 2000 | 1, 3 |
| 38 | *Stenopelmus rufinasus* Gyllenhal, 1835 | 1921 | 1 |
|  | **Dermestidae** |  |  |
| 39 | *Attagenus fasciatus* (Thunberg, 1795) | 1943 | 5 |
| 40 | *Attagenus smirnovi* Zhantiev, 1973 | 1978 | 5 |
| 41 | *Dermestes ater* De Geer, 1774 | 1900 | 5 |
| 42 | *Trogoderma glabrum* (Herbst, 1783) | 1956 | 5 |
|  | **Elateridae** |  |  |
| 43 | *Panspoeus guttatus* Sharp, 1877 | 1981 | 1, 3 |
|  | **Hydrophilidae** |  |  |
| 44 | *Cryptopleurum subtile* Sharp, 1884 | 1958 | 1 |
|  | **Leiodidae** |  |  |
| 45 | *Aglyptinus agathidioides* Blair, 1930 | 1912 | 1, 5 |
|  | **Mycetophagidae** |  |  |
| 46 | *Eulagius filicornis* (Reitter, 1887) | 1995 | 1 |
|  | **Nitidulidae** |  |  |
| 47 | *Pocadius adustus* Reitter, 1888 | 2004 | 1 |
|  | **Ptiliidae** |  |  |
| 48 | *Acrotrichis cognata* (Matthews, 1877) | 1967 | 1 |
| 49 | *Acrotrichis henrici* (Matthews, 1872) | 1966 | 1 |
| 50 | *Acrotrichis insularis* (Maklin, 1852) | 1965 | 1 |
| 51 | *Acrotrichis josephi* (Matthews, 1872) | 1987 | 1 |
| 52 | *Acrotrichis sanctaehelenae* Johnson, 1972 | 1985 | 1 |
| 53 | *Ptinella cavelli* (Broun, 1893) | 1936 | 1 |
| 54 | *Ptinella errabunda* Johnson, 1975 | 1925 | 1 |
| 55 | *Ptinella simsoni* (Matthews, 1878) | 1929 | 1 |
| 56 | *Ptinella taylorae J*ohnson, 1977 | 1967 | 1 |
|  | **Staphylinidae** |  |  |
| 57 | *Hadrognathus longipalpis* (Mulsant and Rey, 1851) | 1989 | 1 |
|  | **Zopheridae** |  |  |
| 58 | *Pycnomerus fuliginosus* Erichson, 1842 | 1962 | 1 |
|  | **Dermaptera** |  |  |
|  | **Anisolabididae** |  |  |
| 59 | *Euborellia annulipes* (Lucas, 1847) | 1920 | 1, 5 |
|  | **Dictyoptera** |  |  |
|  | **Blattellidae** |  |  |
| 60 | *Loboptera decipiens* (Germar, 1817) | 1997 | 3 |
|  | **Rhinotermitidae** |  |  |
| 61 | *Reticulitermes lucifugus* (Rossi, 1792) | 1974 | 1 |
|  | **Diptera** |  |  |
|  | **Agromyzidae** |  |  |
| 62 | *Phytomyza gymnostoma* (Loew, 1858) | 2002 | 3 |
| 63 | *Phytomyza hellebori* Kaltenbach, 1872 | 1997 | 3 |
|  | **Braulidae** |  |  |
| 64 | *Braula schmitzi* Örösi-Pál, 1939 | 1994 | 3 |
|  | **Cecidomyiidae** |  |  |
| 65 | *Aphidoletes abietis* (Kieffer, 1896) | 2005 | 1 |
| 66 | *Blastodiplosis cocciferae* Tavares, 1901 | 2005 | 1 |
| 67 | *Contarinia quinquenotata* (Löw, 1888) | 1985 | 3 |
| 68 | *Corticaria abietorum* Motschulsky, 1867 | 1992 | 1 |
| 69 | *Dasineura abietiperda* (Henschel, 1880) | 2000 | 1 |
| 70 | *Dasineura gleditchiae* (Osten Sacken, 1866) | 1983 | 3 |
| 71 | *Dasineura kellneri* (Henschel, 1875) | 2000 | 1 |
| 72 | *Feltiella acarisuga* (Vallot, 1827) | 1995 | 5 |
| 73 | *Janetiella siskiyou* Felt, 1917 | 1969 | 1 |
| 74 | *Kaltenbachiola strobi* (Winnertz, 1853) | 2000 | 1 |
| 75 | *Psectrosema tamaricis* (Stefani, 1902) | 1922 | 1 |
|  | **Culicidae** |  |  |
| 76 | *Aedes vexans* (Meigen, 1830) | 1930 | 1 |
|  | **Dolichopodidae** |  |  |
| 77 | *Micropygus vagans* Parent, 1933 | 2005 | 1 |
|  | **Drosophilidae** |  |  |
| 78 | *Drosophila busckii* Coquillett, 1901 | 1990 | 1 |
| 79 | *Drosophila hydei* Sturtevant, 1921 | 1990 | 1 |
| 80 | *Drosophila immigrans* Sturtevant, 1921 | 1990 | 1 |
| 81 | *Drosophila melanogaster* Meigen, 1830 | 1990 | 1 |
| 82 | *Drosophila repleta* Wollaston, 1858 | 1990 | 1 |
|  | **Heleomyzidae** |  |  |
| 83 | *Prosopantrum flavifrons* (Tonnoir and Malloch, 1927) | 1991 | 1 |
|  | **Muscidae** |  |  |
| 84 | *Atherigona varia* (Meigen, 1826) | 1998 | 1 |
|  | **Platypezidae** |  |  |
| 85 | *Agathomyia wankowiczii* (Schnabl, 1884) | 1990 | 1 |
|  | **Scathophagidae** |  |  |
| 86 | *Norellia spinipes* (Meigen, 1826) | 1969 | 1 |
|  | **Sciaridae** |  |  |
| 87 | *Bradysia difformis* Frey, 1948 | 2005 | 1 |
|  | **Sphaeroceridae** |  |  |
| 88 | *Thoracochaeta johnsoni* (Spuler, 1925) | 1999 | 1 |
| 89 | *Thoracochaeta seticosta* (Spuler, 1925) | 1999 | 1 |
|  | **Syrphidae** |  |  |
| 90 | *Eumerus tuberculatus* Rondani, 1857 | 1905 | 1 |
|  | **Tachinidae** |  |  |
| 91 | *Catharosia pygmaea* (Fallén, 1815) | 1998 | 1 |
| 92 | *Clytiomya continua* (Panzer, 1798) | 1996 | 1 |
| 93 | *Phasia barbifrons* (Girschner, 1887) | 1993 | 1 |
|  | **Tephritidae** |  |  |
| 94 | *Chetostoma curvinerve* Rondani, 1856 | 1999 | 1 |
| 95 | *Rhagoletis meigenii* (Loew, 1844) | 2005 | 1 |
| 96 | *Tephritis matricariae* (Loew, 1844) | 2000 | 1, 3 |
| 97 | *Tephritis praecox* (Loew, 1844) | 2002 | 1, 3 |
| 98 | *Terellia fuscicornis* (Loew, 1844) | 2000 | 3 |
|  | **Tethinidae** |  |  |
| 99 | *Pelomyia occidentalis* Williston, 1893 | 2000 | 1 |
|  | **Hemiptera** |  |  |
|  | **Adelgidae** |  |  |
| 100 | *Adelges abietis* (Linnaeus 1758) | 1922 | 1 |
| 101 | *Adelges cooleyi* (Gillette, 1907) | 1913 | 1 |
| 102 | *Adelges viridana* Cholodkovsky, 1896 | 1949 | 1 |
| 103 | *Pineus cembrae* (Cholodkovsky, 1888) | 1981 | 3 |
| 104 | *Pineus orientalis* (Dreyfus, 1889) | 1924 | 1 |
| 105 | *Pineus pineoides* Cholodkovsky, 1903 | 1969 | 1 |
| 106 | *Pineus similis* (Gillette, 1907) | 1971 | 1, 3 |
| 107 | *Pineus strobi* (Hartig, 1837) | 1923 | 1 |
|  | **Aleyrodidae** |  |  |
| 108 | *Aleurothrixus floccosus* (Maskell, 1895) | 1986 | 3 |
| 109 | *Bemisia afer* (Priesner and Hosny, 1934) | 1980 | 3 |
|  | **Aphididae** |  |  |
| 110 | *Amphorophora tuberculata* Brown and Blackman, 1985 | 1984 | 1 |
| 111 | *Aphis oenotherae* Oestlund, 1887 | 1992 | 1, 3 |
| 112 | *Aphis spiraecola* Patch, 1914 | 1978 | 1 |
| 113 | *Appendiseta robiniae* (Gillette, 1907) | 1982 | 1, 3 |
| 114 | *Cinara acutirostris* Hille Ris Lambers, 1956 | 1950 | 1 |
| 115 | *Cinara brauni* Börner, 1940 | 1968 | 1 |
| 116 | *Cinara cedri* Mimeur, 1936 | 1971 | 3 |
| 117 | *Cinara confinis* (Koch, 1856) | 1913 | 1 |
| 118 | *Cinara curvipes* (Patch, 1912) | 1999 | 3 |
| 119 | *Cinara escherichi* (Börner, 1950) | 1967 | 1 |
| 120 | *Cinara fresai* Blanchard, 1939 | 1956 | 1 |
| 121 | *Cinara laportei* (Remaudière, 1954) | 1974 | 1, 3 |
| 122 | *Cinara pectinatae* (Nördlinger, 1880) | 1914 | 1 |
| 123 | *Cinara piceicola* (Cholodkovsky, 1896) | 1961 | 1 |
| 124 | *Cinara schimitscheki* Börner, 1940 | 1950 | 1 |
| 125 | *Cinara tujafilina* (Del Guercio, 1909) | 1935 | 1 |
| 126 | *Crypturaphis grassii* Silvestri, 1935 | 1997 | 3, 6 |
| 127 | *Illinoia azaleae* (Mason, 1925) | 1950 | 1 |
| 128 | *Illinoia lambersi* (MacGillivray, 1960) | 1971 | 1, 3 |
| 129 | *Illinoia liriodendri* (Monell, 1879) | 2003 | 3 |
| 130 | *Impatientinum asiaticum* Nevsky, 1929 | 1983 | 1 |
| 131 | *Macrosiphum albifrons* Essig, 1911 | 1981 | 1, 3 |
| 132 | *Mindarus abietinus* Koch, 1857 | 1904 | 1 |
| 133 | *Mindarus obliquus* (Cholodkovsky, 1896) | 1967 | 1 |
| 134 | *Myzus hemerocallis* Takahashi, 1921 | 2000 | 3 |
| 135 | *Myzus varians* Davidson, 1912 | 1970 | 1, 3 |
| 136 | *Nearctaphis bakeri* (Cowen, 1895) | 1969 | 1 |
| 137 | *Prociphilus fraxini* (Fabricius, 1777) | 1970 | 1, 3 |
| 138 | *Sitobion ptericolens* (Patch,1919) | 1972 | 1, 3 |
| 139 | *Stagona pini* (Burmeister, 1835) | 1915 | 1 |
| 140 | *Takecallis arundinariae* (Essig, 1917) | 1999 | 3 |
| 141 | *Takecallis taiwanus* (Takahashi, 1926) | 1999 | 3 |
| 142 | *Tinocallis nevskyi* Remaudière, Quednau and Heie, 1988 | 1995 | 1, 3 |
| 143 | *Trichosiphonaphis polygonifoliae* (Shinji, 1944) | 1999 | 3 |
| 144 | *Uroleucon erigeronense* (Thomas, 1878) | 1973 | 1, 3 |
| 145 | *Utamphorophora humboldti* (Essig, 1941) | 1974 | 1, 3 |
| 146 | *Wahlgreniella nervata* (Gillette, 1908) | 1973 | 1, 3 |
|  | **Asterolecaniidae** |  |  |
| 147 | *Russellaspis pustulans* (Cockerell, 1892) | 1982 | 3 |
|  | **Cicadellidae** |  |  |
| 148 | *Graphocephala fennahi* Young, 1977 | 1933 | 1 |
| 149 | *Iassus scutellaris* (Fieber, 1868) | 1978 | 1, 3 |
| 150 | *Idiocerus ustulatus* (Mulsant and Rey, 1855) | 1991 | 3 |
| 151 | *Placotettix taeniatifrons* (Kirschbaum, 1868) | 1951 | 1 |
|  | **Coccidae** |  |  |
| 152 | *Eulecanium excrescens* (Ferris, 1920) | 1998 | 3 |
| 153 | *Pulvinaria hydrangeae* Steinweden, 1946 | 1987 | 3 |
| 154 | *Pulvinaria regalis* Canard, 1968 | 1964 | 1 |
| 155 | *Pulvinariella mesembryanthemi* (Vallot, 1830) | 1975 | 1 |
|  | **Diaspididae** |  |  |
| 156 | *Acutaspis umbonifera* (Newstead, 1920) | 1970 | 3 |
| 157 | *Aonidia lauri* (Bouché, 1833) | 1990 | 3 |
| 158 | *Eulepidosaphes pyriformis* (Maskell, 1897) | 1975 | 1, 3 |
| 159 | *Fiorinia externa* Ferris, 1942 | 1980 | 3 |
| 160 | *Leucaspis podocarpi* Green, 1929 | 1975 | 3 |
| 161 | *Odonaspis greenii* Cockerell, 1902 | 1970 | 3 |
| 162 | *Pseudaulacaspis dubia* (Maskell, 1882) | 1994 | 3 |
|  | **Homotomidae** |  |  |
| 163 | *Homotoma ficus* (Linnaeus, 1758) | 1990 | 1 |
|  | **Lygaeidae** |  |  |
| 164 | *Nysius senecionis* (Schilling, 1829) | 1992 | 3 |
| 165 | *Orsillus depressus* Dallas, 1852 | 1987 | 1, 3, 7 |
| 166 | *Peritrechus gracilicornis* Puton, 1877 | 1977 | 3 |
|  | **Margarodidae** |  |  |
| 167 | *Icerya purchasi* Maskell, 1879 | 1996 | 1, 3 |
|  | **Miridae** |  |  |
| 168 | *Campylomma annulicornis* (Signoret, 1865) | 1978 | 3 |
| 169 | *Deraeocoris flavilinea* (Costa, 1862) | 1996 | 3 |
| 170 | *Dichrooscytus gustavi* Josifov, 1981 | 1988 | 7 |
| 171 | *Hypseloecus visci* (Puton, 1888) | 2003 | 3 |
| 172 | *Placochilus seladonicus* (Fallén, 1807) | 1977 | 3 |
| 173 | *Tupiocoris rhododendri* (Dolling, 1972) | 1971 | 1, 3, 7 |
| 174 | *Tuponia brevirostris* Reuter, 1883 | 2001 | 7 |
| 175 | *Tuponia mixticolor* (Costa, 1862) | 1979 | 1, 3, 7 |
|  | **Pemphigidae** |  |  |
| 176 | *Paracolopha morrisoni* (Baker,1919) | 1990 | 3 |
|  | **Pentatomidae** |  |  |
| 177 | *Eurydema ornata* (Linnaeus, 1758) | 1997 | 3 |
| 178 | *Nezara viridula* (Linnaeus, 1758) | 2003 | 1, 3, 7 |
| 179 | *Pentaloma nigra* Ferris, 1942 | 1996 | 3 |
|  | **Phylloxeridae** |  |  |
| 180 | *Moritziella corticalis* (Kaltenbach, 1867) | 1970 | 1, 3 |
|  | **Pseudococcidae** |  |  |
| 181 | *Geococcus coffeae* Green, 1933 | 1996 | 3 |
| 182 | *Phenacoccus defectus* Ferris, 1950 | 1990 | 3 |
| 183 | *Planococcus vovae*  (Nassonov, 1908) | 1982 | 3 |
| 184 | *Rhizoecus aloes* Williams and Pellizzari (1997) | 1996 | 3 |
| 185 | *Spilococcus cactearum* McKenzie, 1960 | 1988 | 3 |
| 186 | *Trochiscococcus speciosus* (De Lotto, 1961) | 1996 | 3 |
| 187 | *Vryburgia amaryllidis* (Bouché, 1837) | 1970 | 3 |
| 188 | *Vryburgia brevicruris* (McKenzie, 1960) | 1978 | 3 |
|  | **Psyllidae** |  |  |
| 189 | *Acizzia uncatoides* (Ferris and Klyver, 1932) | 1990 | 3 |
| 190 | *Cacopsylla fulguralis* (Kuwayama, 1908) | 2002 | 1, 3 |
| 191 | *Ctenarytaina eucalypti* (Maskell, 1895) | 1922 | 1 |
| 192 | *Floria variegata* (Löw, 1881) | 1978 | 1 |
| 193 | *Livilla variegata* (Löw, 1881) | 1978 | 3 |
|  | **Rhopalidae** |  |  |
| 194 | *Brachycarenus tigrinus* (Schilling, 1829) | 2003 | 3 |
| 195 | *Liorhyssus hyalinus* (Fabricius, 1794) | 1996 | 3 |
| 196 | *Stictopleurus abutilon* (Rossi, 1790) | 1996 | 3 |
| 197 | *Stictopleurus punctatonervosus* (Goeze, 1778) | 1998 | 3 |
|  | **Tingidae** |  |  |
| 198 | *Corythucha ciliata* (Say, 1832) | 2005 | 7 |
| 199 | *Stephanitis rhododendri* Horvath, 1905 | 1901 | 1, 7 |
| 200 | *Stephanitis takeyai* Drake and Maa, 1955 | 1995 | 1, 3, 7 |
|  | **Triozidae** |  |  |
| 201 | *Trioza vitreoradiata*  (Maskell, 1879) | 1993 | 1, 3 |
|  | **Hymenoptera** |  |  |
|  | **Argidae** |  |  |
| 202 | *Arge berberidis* Schrank, 1802 | 2000 | 3 |
|  | **Cynipidae** |  |  |
| 203 | *Andricus corruptrix* (Schlechtendal, 1870) | 1972 | 1, 3 |
| 204 | *Andricus grossulariae* Giraud, 1859 | 2000 | 1, 3 |
| 205 | *Andricus lignicola* (Hartig, 1840) | 1972 | 1, 3 |
| 206 | *Andricus quercuscalicis* (Burgsdorf, 1783) | 1961 | 1 |
|  | **Diprionidae** |  |  |
| 207 | *Gilpinia hercyniae* (Hartig, 1837) | 1906 | 1 |
|  | **Formicidae** |  |  |
| 208 | *Wasmannia auropunctata* (Roger, 1863) | 1907 | 4 |
|  | **Pamphiliidae** |  |  |
| 209 | *Cephalcia lariciphila* (Wachtl, 1898) | 1953 | 1 |
|  | **Siricidae** |  |  |
| 210 | *Tremex columba* (Linnaeus, 1763) | 1957 | 1 |
|  | **Tenthredinidae** |  |  |
| 211 | *Aphelonyx cerricola* (Giraud, 1859) | 1993 | 1, 3 |
| 212 | *Nematus spiraeae* Zaddach, 1883 | 1924 | 1 |
| 213 | *Pachynematus imperfectus* (Zaddach, 1876) | 1929 | 1 |
| 214 | *Pristiphora erichsonii* (Hartig, 1837) | 1906 | 1 |
| 215 | *Pristiphora leucopus* (Hellén, 1948) | 2004 | 1 |
|  | **Torymidae** |  |  |
| 216 | *Megastigmus pinus* Parfitt, 1857 | 1953 | 1 |
| 217 | *Megastigmus spermotrophus* Wachtl, 1893 | 1906 | 1 |
|  | **Lepidoptera** |  |  |
|  | **Blastobasidae** |  |  |
| 218 | *Blastobasis decolorella* Wollaston, 1858 | 1946 | 1 |
| 219 | *Blastobasis phycidella* (Zeller, 1839) | 1998 | 1 |
| 220 | *Blastobasis vittata* (Wollaston, 1858) | 1917 | 1 |
|  | **Castniidae** |  |  |
| 221 | *Paysandisia archon* (Burmeister, 1879) | 2002 | 8 |
|  | **Coleophoridae** |  |  |
| 222 | *Coleophora aestuariella* Bradley, 1984 | 1981 | 3 |
| 223 | *Coleophora fuscicornis* Zeller, 1847 | 1973 | 3 |
| 224 | *Coleophora linosyridella* Fuchs, 1880 | 1978 | 3 |
|  | **Douglasiidae** |  |  |
| 225 | *Tinagma balteolella* (Fischer von Röslerstamm, 1841) | 1975 | 3 |
|  | **Gelechiidae** |  |  |
| 226 | *Anarsia lineatella* Zeller, 1839 | 1959 | 1 |
| 227 | *Athrips rancidella* (Herrich-Schäffer, 1854) | 1971 | 3 |
| 228 | *Coleotechnites piceaella* (Kearfott, 1903) | 1952 | 1 |
| 229 | *Gelechia sabinellus* (Zeller, 1839) | 1971 | 3 |
| 230 | *Gelechia senticetella* (Staudinger, 1859) | 1988 | 1, 3 |
| 231 | *Monochroa moyses* Uffen, 1991 | 1971 | 3 |
| 232 | *Monochroa niphognatha* (Gozmány, 1953) | 1984 | 3 |
|  | **Geometridae** |  |  |
| 233 | *Eupithecia phoeniceata* (Rambur, 1834) | 1959 | 1 |
| 234 | *Eupithecia ultimaria* Boisduval, 1840 | 1989 | 3 |
| 235 | *Peribatodes secundaria* (Denis and Schiffermüller, 1775) | 1981 | 1, 3 |
| 236 | *Thera cupressata* (Geyer, 1831) | 1984 | 3 |
|  | **Gracillariidae** |  |  |
| 237 | *Caloptilia azaleella* (Brants, 1913) | 1936 | 1 |
| 238 | *Caloptilia rufipennella* (Hübner, 1796) | 1970 | 3 |
| 239 | *Cameraria ohridella* Deschka and Dimic, 1986 | 2002 | 1, 3 |
| 240 | *Phyllocnistis xenia* Hering, 1936 | 1974 | 3 |
| 241 | *Phyllonorycter leucographella* (Zeller, 1850) | 1989 | 3 |
| 242 | *Phyllonorycter platani* (Staudinger, 1870) | 1990 | 1, 3 |
| 243 | *Phyllonorycter strigulatella* (Lienig and Zeller, 1846) | 1928 | 1 |
|  | **Lycaenidae** |  |  |
| 244 | *Cacyreus marshalli* Butler, 1898 | 1997 | 9 |
|  | **Nepticulidae** |  |  |
| 245 | *Ectoedemia erythrogenella* (Joannis, 1908) | 1973 | 3 |
| 246 | *Ectoedemia hannoverella* (Glitz, 1872) | 2003 | 3 |
| 247 | *Ectoedemia heringella* (Mariani, 1939) | 2002 | 1, 3 |
| 248 | *Ectoedemia sericopeza* (Zeller, 1839) | 1975 | 3 |
|  | **Noctuidae** |  |  |
| 249 | *Dryobota labecula* (Esper, 1788) | 1999 | 3 |
| 250 | *Hadena compta* (Denis and Schiffermüller, 1775) | 1948 | 1 |
| 251 | *Hecatera dysodea* (Denis and Schiffermüller 1775) | 1997 | 3 |
| 252 | *Hypena obsitalis* (Hübner, 1813) | 1990 | 3 |
|  | **Notodontidae** |  |  |
| 253 | *Clostera anachoreta* (Denis and Schiffermüller, 1775) | 1979 | 3 |
|  | **Oecophoridae** |  |  |
| 254 | *Tachystola acroxantha* (Meyrick, 1885) | 1908 | 1 |
|  | **Prodoxidae** |  |  |
| 255 | *Lampronia flavimitrella* (Hübner, 1817) | 1974 | 3 |
|  | **Pterophoridae** |  |  |
| 256 | *Stenoptilia millieridactyla* (Bruand, 1861) | 1969 | 1 |
|  | **Pyralidae** |  |  |
| 257 | *Crambus leucoschalis* Hampson, 1898 | 1920 | 1 |
| 258 | *Dioryctria schuetzeella* Fuchs, 1899 | 1980 | 1, 3 |
| 259 | *Dioryctria sylvestrella* (Ratzeburg, 1840) | 1999 | 3 |
| 260 | *Duponchelia fovealis* Zeller, 1847 | 1996 | 3 |
| 261 | *Evergestis limbata* (Linnaeus, 1767) | 1993 | 3 |
| 262 | *Haimbachia cicatricella* (Hübner, 1824) | 1999 | 3 |
| 263 | *Maruca testulalis* Geyer, 1832 | 1979 | 1 |
| 264 | *Sceliodes laisalis* (Walker, 1859) | 1973 | 1 |
| 265 | *Sciota adelphella* (Fischer von Röslerstamm, 1836) | 1992 | 3 |
| 266 | *Sclerocona acutellus* (Eversmann, 1842) | 1988 | 1 |
| 267 | *Vitula biviella* (Zeller, 1848) | 1997 | 3 |
|  | **Scythrididae** |  |  |
| 268 | *Scythris inspersella* (Hübner, 1817) | 1977 | 3 |
|  | **Tineidae** |  |  |
| 269 | *Psychoides filicivora* (Meyrick, 1937) | 1940 | 1 |
| 270 | *Tinea pallescentella* Stainton, 1851 | 1951 | 1 |
|  | **Tischeriidae** |  |  |
| 271 | *Emmetia heinemanni* (Wocke, 1871) | 1984 | 3 |
| 272 | *Epiphyas postvittana* (Walker, 1863) | 1936 | 1 |
|  | **Tortricidae** |  |  |
| 273 | *Adoxophyes orana* (Fischer von Röslerstamm, 1834) | 1950 | 1 |
| 274 | *Cacoecimorpha pronubana* (Hübner, 1799) | 1905 | 1 |
| 275 | *Cochylis molliculana* Zeller, 1847 | 1993 | 3 |
| 276 | *Crocidosema plebejana* Zeller, 1847 | 1900 | 1 |
| 277 | *Cryptoblabes gnidiella* (Millière, 1867) | 1936 | 1 |
| 278 | *Cydia conicolana* (Heylaerts, 1874) | 1930 | 1 |
| 279 | *Cydia illutana* (Herrich-Schäffer, 1851) | 1975 | 1, 3 |
| 280 | *Cydia medicaginis* (Kuznetsov, 1962) | 1970 | 3 |
| 281 | *Cydia milleniana* (Adamczewski, 1967) | 1944 | 1 |
| 282 | *Cydia pactolana* (Zeller, 1840) | 1965 | 1 |
| 283 | *Eucosma metzneriana* (Treitschke, 1830) | 1998 | 3 |
|  | **Yponomeutidae** |  |  |
| 284 | *Argyresthia cupressella* Walsingham, 1890 | 1997 | 3 |
| 285 | *Argyresthia trifasciata* Staudinger, 1871 | 1982 | 1, 3 |
|  | **Neuroptera** |  |  |
|  | **Coniopterygidae** |  |  |
| 286 | *Aleuropteryx juniperi* Ohm, 1968 | 1966 | 1 |
|  | **Orthoptera** |  |  |
| 287 | *Meconema meridionale* Costa, 1860 | 2001 | 1 |
|  | **Phasmatodea** |  |  |
| 288 | *Acanthoxyla prasina geisovii* (Kaup, 1866) | 1907 | 1 |
| 289 | *Acanthoxyla prasina inermis* Salmon, 1955 | 1929 | 1 |
| 290 | *Clitarchus hookeri* (White, 1846) | 1910 | 1 |
|  | **Psocoptera** |  |  |
|  | **Trogiidae** |  |  |
| 291 | *Lepinotus patruelis* Pearman, 1931 | 2004 | 5 |
|  | **Thysanoptera** |  |  |
|  | **Phlaeothripidae** |  |  |
| 292 | *Hoplothrips unicolor* (Vuillet, 1914) | 1939 | 5 |
| 293 | *Suocerathrips lingui*s Mound and Marullo, 1994 | 1994 | 5 |
|  | **Thripidae** |  |  |
| 294 | *Echinothrips americanus* Morgan, 1913 | 1995 | 3 |
| 295 | *Frankliniella occidentalis* (Pergande, 1895) | 1986 | 3 |
| 296 | *Thrips palmi* Karny, 1925 | 2000 | 10 |

List of FRD information sources:

1. Hill M, Baker R, Broad G, Chandler PJ, Copp GH, et al. (2005) Audit of non-native species in England. English Nature Research Reports No. 662. U.K.: English Nature.
2. EPPO. (2008) Situation of *Anoplophora chinensis* in the United Kingdom. *EPPO reporting service*. Paris, 2008-05-01.
3. Smith RM, Baker RHA, Malumphy CP, Hockland S, Hammon RP, et al. (2007) Recent non-native invertebrate plant pest establishments in Great Britain: origins, pathways, and trends. Agricultural and Forest Entomology 9: 307–326.
4. Global Invasive Species Database (http://www.issg.org/database). Latest accessed date: 28 December, 2010.
5. DAISIE. European Invasive Alien Species Gateway (http://www.europe-aliens.org). Latest accessed date: 28 December, 2010.
6. EPPO. (1998) Finding of *Crypturaphis grassii* in United Kingdom. *EPPO reporting service*. Paris, 1998-09-01.
7. Rabitsch W (2008) Alien true bugs of Europe (Insecta: Hemiptera: Heteroptera). Zootaxa 1827: 1–44.
8. EPPO. (2003) First report of *Paysandisia archon* in United Kingdom. *EPPO reporting service*. Paris, 2003-08-01.
9. EPPO. (1998) Possibilities of biological control of *Cacyreus marshalli.* *EPPO reporting service.* Paris, 1998-08-01.
10. EPPO. (2001) *Thrips palmi* reported in United Kingdom. *EPPO reporting service.* Paris, 2001-01-01.
