# Supplementary material for: Does Global Warming Increase Establishment Rates of Invasive Alien Species? A Centurial Time Series Analysis
Source: PLoS One. 2011 Sep 8;6(9):e24733. doi: 10.1371/journal.pone.0024733 (PMC3169637; doi:10.1371/journal.pone.0024733)
Supplement: Table S3 — List of invasive and noninvasive alien scolytines and their first-recorded dates of establishment in the contiguous United States during 1900–2005 (inclusive). (DOC) [file pone.0024733.s003.doc]

# Table S3. List of alien scolytines and their first-recorded dates (FRDs) in the contiguous United States during 1900–2005 (inclusive). All FRDs were derived from Haack RA, Rabaglia RJ (2011) Exotic bark and ambrosia beetles (Coleoptera: Curculionidae: Scolytinae) in the United States: potential and current invaders. In Peña JE (ed.) Potential invasive pests of agricultural crop species. CAB International, Wallingford, UK. (In press).

|  | **Tribe** | **FRD** |
| --- | --- | --- |
|  | **Species** |
|  | **Hylastini** |  |
| 1 | *Hylastes opacus* Erichson, 1836 | 1987 |
| 2 | *Hylurgops palliatus* (Gyllenhal, 1813) | 2001 |
|  | **Tomicini** |  |
| 3 | *Hylurgus ligniperda* (Fabricius, 1787) | 1994 |
| 4 | *Tomicus piniperda* (Linnaeus, 1758) | 1991 |
|  | **Phloeosinini** |  |
| 5 | *Phloeosinus armatus* Reitter, 1887 | 1992 |
|  | **Scolytini** |  |
| 6 | *Scolytus multistriatus* (Marsham, 1802) | 1909 |
| 7 | *Scolytus schevyrewi* Semenov, 1902 | 1994 |
|  | **Ipini** |  |
| 8 | *Orthotomicus erosus* (Wollaston, 1857) | 2004 |
| 9 | *Pityogenes bidentatus* (Herbst, 1784) | 1988 |
|  | **Dryocoetini** |  |
| 10 | *Coccotrypes advena* Blandford, 1894 | 1956 |
| 11 | *Coccotrypes carpophagus* (Hornung, 1842) | 1926 |
| 12 | *Coccotrypes cyperi* (Beeson, 1929) | 1934 |
| 13 | *Coccotrypes dactyliperda* (Fabicius, 1801) | 1915 |
| 14 | *Coccotrypes distinctus* (Motschulsky, 1866) | 1939 |
| 15 | *Coccotrypes rhizophorae* (Hopkins, 1915) | 1910 |
| 16 | *Coccotrypes rutschuruensis* Eggers, 1940 | 1992 |
| 17 | *Coccotrypes vulgaris* (Eggers, 1940) | 1985 |
| 18 | *Dryoxylon onoharaensum* (Murayama, 1934) | 1982 |
|  | **Premnobini** |  |
| 19 | *Premnobius cavipennis* Eichhoff, 1878 | 1939 |
|  | **Xyleborini** |  |
| 20 | *Ambrosiodmus lewisi* (Blandford, 1894) | 1990 |
| 21 | *Ambrosiodmus rubricollis* (Eichhoff, 1875) | 1942 |
| 22 | *Ambrosiophilus atratus* (Eichhoff, 1875) | 1988 |
| 23 | *Anisandrus maiche* Stark, 1936 | 2005 |
| 24 | *Cnestus mutilatus* (Blandford, 1894) | 1999 |
| 25 | *Cyclorhipidion californicus* (Wood) | 1944 |
| 26 | *Cyclorhipidion pelliculosum* Hagedorn, 1912 | 1987 |
| 27 | *Euwallacea fornicatus* (Eichhoff, 1868) | 2002 |
| 28 | *Euwallacea validus* (Eichhoff, 1875) | 1976 |
| 29 | *Wallacellus similis* (Ferrari, 1867) | 2002 |
| 30 | *Xyleborinus alni* Niisima, 1909 | 1996 |
| 31 | *Xyleborinus saxesenii* (Ratzeburg, 1837) | 1911 |
| 32 | *Xyleborus glabratus* Eichhoff, 1877 | 2002 |
| 33 | *Xyleborus pfeilii* (Ratzeburg, 1837) | 1992 |
| 34 | *Xyleborus seriatus* Blandford, 1894 | 2005 |
| 35 | *Xylosandrus compactus* (Eichhoff, 1875) | 1941 |
| 36 | *Xylosandrus crassiusculus* (Motschulsky, 1866) | 1974 |
| 37 | *Xylosandrus germanus* (Blandford, 1894) | 1931 |
|  | **Cryphalini** |  |
| 38 | *Hypocryphalus mangiferae* (Stebbing, 1914) | 1949 |
| 39 | *Hypothenemus areccae* (Hornung, 1842) | 1960 |
| 40 | *Hypothenemus birmanus* (Eichhoff, 1878) | 1951 |
| 41 | *Hypothenemus brunneus* (Hopkins, 1915) | 1904 |
| 42 | *Hypothenemus javanus* (Eggers, 1908) | 1977 |
| 43 | *Hypothenemus obscurus* (Fabricius, 1801) | 1977 |
| 44 | *Hypothenemus setosus* (Eichhoff, 1868) | 1982 |
