# Supplementary material for: Does Global Warming Increase Establishment Rates of Invasive Alien Species? A Centurial Time Series Analysis
Source: PLoS One. 2011 Sep 8;6(9):e24733. doi: 10.1371/journal.pone.0024733 (PMC3169637; doi:10.1371/journal.pone.0024733)
Supplement: Table S4 — Minimum and maximum values for the response and explanatory variables used in the present study by country. (DOC) [file pone.0024733.s004.doc]

**Table S4.** Minimum and maximum values for the response and explanatory variables used in the present study by country.

| **Variable (Unit)** | **China** | **United Kingdom** | **United States** |
| --- | --- | --- | --- |
| **Establishment rate of invasive aliens species (species/year)** | | | |
| Annual series |  |  |  |
| Study period | 1900–2005 | 1900–2005 | 1900–2005 |
| Min | 0 | 0 | 0 |
| Max | 4 | 13 | 3 |
| 11-year moving average series |  |  |  |
| Study period | 1905–2000 | 1905–2000 | 1905–2000 |
| Min | 0.091 | 0.727 | 0 |
| Max | 1.545 | 7.727 | 1.091 |
| **Change in average annual surface air temperature (°C/year)** | | | |
| Reference period | 1960–1990 | 1960–1990 | 1960–1990 |
| Annual series |  |  |  |
| Study period | 1900–2005 | 1900–2005 | 1900–2005 |
| Min | -1.00 | -1.00 | -1.04 |
| Max | 1.45 | 1.16 | 1.29 |
| 11-year moving average series |  |  |  |
| Study period | 1905–2000 | 1905–2000 | 1905–2000 |
| Min | -0.576 | -0.360 | -0.300 |
| Max | 0.899 | 0.845 | 0.645 |
| **Value of merchandise imports (Millions US dollar/year)** | | | |
| Annual series |  |  |  |
| Study period | 1950–2005 | 1950–2005 | 1950–2005 |
| Min | 580 | 7305 | 9631 |
| Max | 659,953 | 482,783 | 1,735,060 |
| 11-year moving average series |  |  |  |
| Study period | 1955–2000 | 1955–2000 | 1955–2000 |
| Min | 1720 | 9792 | 11,599 |
| Max | 283,362 | 345,190 | 1,154,353 |
